# Unexpected early migration of a patent foramen ovale occluder

**DOI:** 10.1007/s00508-021-01894-z

**Published:** 2021-06-15

**Authors:** Johannes Mair, Silvana Müller, Axel Bauer

**Affiliations:** Department of Internal Medicine III—Cardiology and Angiology, Medical University Innsbruck, Anichstraße 35, 6020 Innsbruck, Austria

A Figulla® Flex II 23/25 mm patent foramen ovale (PFO) occluder (Occlutech®, Jena, Germany) was deployed across the atrial septum. Correct positioning was confirmed by fluoroscopy demonstrating the “pacman” sign (Fig. 1a arrow; the septum secundum is lying between both discs) without residual shunt in angiography via the delivery sheath (Fig. 1a), transesophageal echocardiography (TEE) (Fig. 1b, the septum primum and secundum are lying between both discs without partial deployment of its right atrial disc within the PFO tunnel; delivery system marked with arrow), and by stable position on repeated bidirectional “wiggle” maneuvers (supplementary video 1). Despite passing these checks it slipped unexpectedly from the atrial septum and embolized to the proximal abdominal aorta on intense coughing after its release. The PFO occluder migration could be successfully managed by percutaneous retrieval (Fig. 1c, see supplementary video 2). Careful PFO reassessment revealed a large, high-risk, long-tunnel PFO (length 21 mm, width 6 mm, septum secundum thickness 6 mm) with a hypermobile atrial septum (septal excursion 10 mm) and a large Eustachian valve (Fig. 1d, arrow). A larger, somewhat less compliant Amplatzer™ 30-mm PFO occluder (Abbott®, Vienna, Austria) was deployed across the PFO and released in the correct position (Fig. 1e) despite a single dislocation of its right atrial disc into the PFO tunnel upon a vigorous push (Fig. 1f, supplementary video 3), because this device was stable afterwards with several repeat “push and pull” maneuvers and a 35 mm PFO occluder was deemed to be too large in this patient considering the risk of erosion and residual shunt. There was no residual shunt in a follow-up TEE after 7 months. The importance of careful assessment of true PFO tunnel length in preprocedural TEE for device size selection is stressed. Other anatomical predisposing factors (atrial septal aneurysm, a > 10 mm thick septum secundum) for occluder migration were not present in this patient [1–3].

**Fig. 1:**
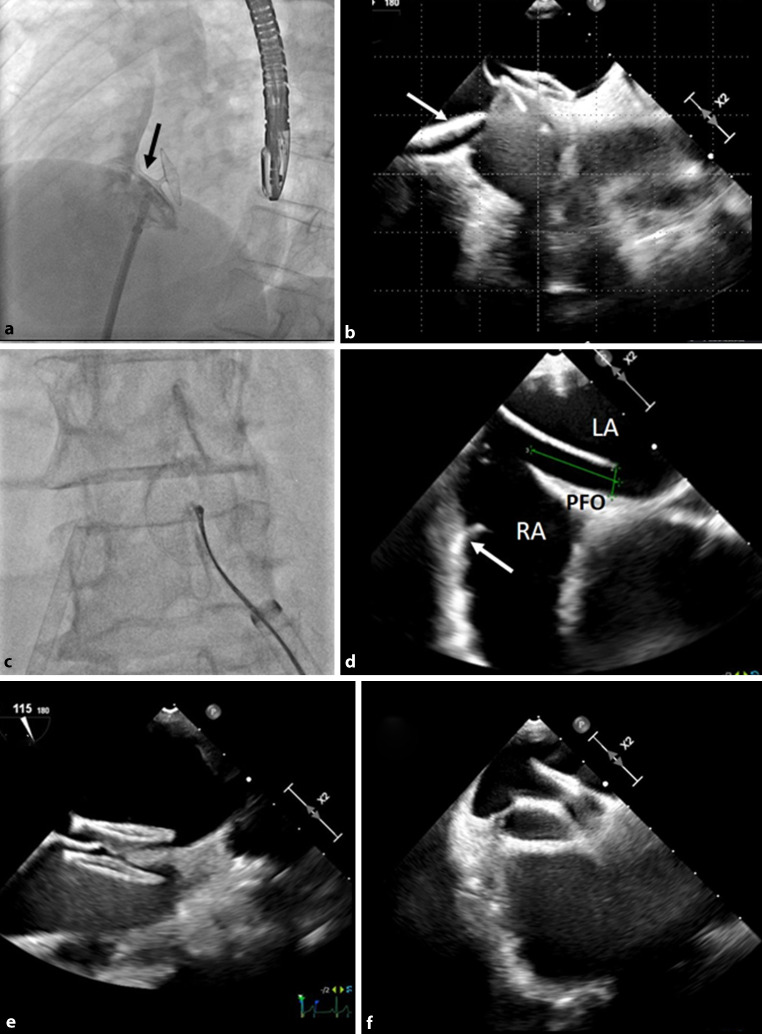
Complicated patent foramen ovale (PFO) occlusion: Figulla® 23/25 mm occluder before release (**a**, **b**); migrated occluder retrieval (**c**); long-tunnel PFO (**d**); Amplatzer® 30 mm PFO occluder before release in correct position (**e**) and with dislocated right atrial disc upon a vigorous push (**f**)

## Supplementary Information

Supplementary video 1: Fluoroscopy of a vigorous “wiggle” test before release of the Occlutech® Figulla® Flex II 23/25 mm PFO occluder. The occluder remained in stable position while still being connected to the delivery system.

Supplementary video 2: Successful interventional retrieval of the embolized Occlutech® Figulla® Flex II 23/25 mm patent foramen ovale occluder through a 18 French 30 cm long femoral sheath using a 35 mm goose neck snare kit

Supplementary video 3: Transesophageal echocardiogram of a vigorous push of the Amplatzer™ 30 mm patent foramen ovale occluder. The right atrial disc migrated into the tunnel upon this vigorous push, which was also the obvious cause of the embolization of the 25/23 mm Occlutech® Figulla II® occluder.

## Abbreviations

LA: Left atrium
PFO: Patent foramen ovale
RA: Right atrium
